# Coastal Fishermen as Lifesavers While Sailing at High Speed: A Crossover Study

**DOI:** 10.1155/2018/2747046

**Published:** 2018-04-24

**Authors:** Ramón Fungueiriño-Suárez, Roberto Barcala-Furelos, Marta González-Fermoso, Santiago Martínez-Isasi, Felipe Fernández-Méndez, Violeta González-Salvado, Rubén Navarro-Patón, Antonio Rodríguez-Núñez

**Affiliations:** ^1^REMOSS Research Group, University of Vigo, Pontevedra, Spain; ^2^Faculty of Education and Sport Sciences, University of Vigo, Pontevedra, Spain; ^3^Complejo Hospitalario de Pontevedra, Pontevedra, Spain; ^4^Faculty of Nursing and Podiatry, Universidade da Coruña, A Coruña, Spain; ^5^University School of Nursing, University of Vigo, Pontevedra, Spain; ^6^CLINURSID Investigation Group, University of Santiago de Compostela, Santiago de Compostela, Spain; ^7^Cardiology Department, University Clinical Hospital of Santiago, Universidade de Santiago de Compostela, Santiago de Compostela, Spain; ^8^Faculty of Education, University of Santiago de Compostela, Lugo, Spain; ^9^Pediatric Emergency and Critical Care Division, Hospital Clínico Universitario de Santiago de Compostela, Santiago de Compostela, Spain

## Abstract

**Purpose:**

Starting basic cardiopulmonary resuscitation (CPR) early improves survival. Fishermen are the first bystanders while at work. Our objective was to test in a simulated scenario the CPR quality performed by fishermen while at port and while navigating at different speeds.

**Methods:**

Twenty coastal fishermen were asked to perform 2 minutes of CPR (chest compressions and mouth-to-mouth ventilations) on a manikin, in three different scenarios: (A) at port on land, (B) on the boat floor sailing at 10 knots, and (C) sailing at 20 knots. Data was recorded using quality CPR software, adjusted to current CPR international guidelines.

**Results:**

The quality of CPR (QCPR) was significantly higher at port (43% ± 10) than sailing at 10 knots (30% ± 15; *p* = 0.01) or at 20 knots (26% ± 12; *p* = 0.001). The percentage of ventilation that achieved some lung insufflation was also significantly higher when CPR was done at port (77% ± 14) than while sailing at 10 knots (59% ± 18) or 20 knots (57% ± 21) (*p* = 0.01).

**Conclusion:**

In the event of drowning or cardiac arrest on a small boat, fishermen should immediately start basic CPR and navigate at a relatively high speed to the nearest port if the sea conditions are safe.

## 1. Introduction

The 2015 European Resuscitation Council (ERC) Guidelines for Resuscitation include basic cardiopulmonary resuscitation (CPR) in special circumstances of drowning [1]. In such cases, prompt and effective actions by lay responders as well as trained rescuers make the difference between life and death [2].

It is known that fishing is one of the most dangerous occupations [3, 4] due to the working conditions, the place where work is done, and the delay in healthcare assistance. In Spain, during the period of 2008–2013, there were more than 100 maritime accidents on fishing vessels. The main causes of death from these incidents were drowning, heart attack, hypothermia, severe/fatal injuries, asphyxia, and burns. In many of these events, CPR by fishermen offshore would be necessary [5].

Consequently, fishermen should receive regular first aid training and periodic retraining about CPR, which would provide them with effective knowledge and skills to handle a maritime emergency.

However, there are some practical questions about resuscitation at sea that remain unanswered: In case of drowning or cardiac arrest on board, should fishermen immediately start CPR or should they “scoop and run” to the nearest land? Is it feasible and effective to perform CPR while navigating to land? How is CPR quality (QCPR) influenced by sailing speed while being done on small fishing boats? Is it possible to perform basic CPR while sailing at a high speed?

Some preliminary studies on the subject have reported promising results [6–9] but, to the best of our knowledge, this is the first time that the sailing speed influence on QCPR by fishermen has been considered.

The aim of this pilot study was to evaluate the competence of fishermen in the execution of CPR on a small fishing boat while at port and during two regular navigation speeds.

## 2. Materials and Methods

### 2.1. Design

A quasi-experimental study with inshore fishermen (*n* = 20) was approved by the Ethics Committee of the Faculty of Sports Sciences (University of Vigo, Spain), respecting the ethical principles of the Helsinki Convention. Each participant authorized, by informed consent, the transfer of data necessary for this research.

Subjects were active professional fishermen, belonging to Rianxo's (A Coruña, Spain) Fishermen Association. They received first aid training in the last two years and they were refreshed in basic CPR according to recommendations of the ERC 2015 with real-time audiovisual feedback equipment just before the current study [10]. The Spanish Legislation of the Ministry of Employment and Social Affairs Maritime Agency for Maritime Occupations requires that retraining CPR certification is necessary every five years [11]. These is supported by the Health Protection and Medical Care for Seafarers Convention of 1987 [12] and STCW Convention of 1995 [13]. Different CPR courses are available to uphold the law, a basic one which lasts eight hours or an advanced one which lasts sixteen hours. Both courses include basic life support.

**Figure 1:**
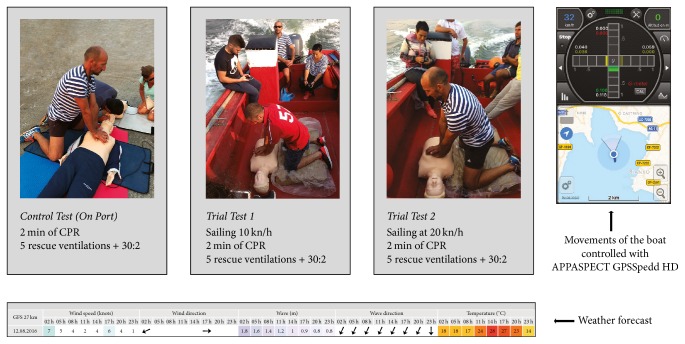
CPR scenarios (baseline test, at port on land, sailing at 10 knots, and sailing at 20 knots), weather forecast, and application (app) movement control.

CPR performance was tested for a 2-minute simulation on a manikin in 3 scenarios: baseline test, at port on land (A), and two tests during sailing with the manikin placed on the boat floor, one at 10-knot speed (B) and the other at 20 knots (C) (Figure 1). At the end of each trial, fishermen were asked about the subjective level of fatigue and self-rated QCPR. Between each test, participants had 30 minutes of rest to avoid the influence of fatigue.

### 2.2. Variables (Figure 2)

The recorded anthropometric variables were weight and height. QCPR variables (chest compressions and mouth-to-mouth ventilations) were recorded using the Laerdal Resusci Anne manikin with SimPad device (Stavanger, Norway) which also allows real-time feedback. It was configured according to the 2015 European Resuscitation Council (ERC) Guidelines [14].

### 2.3. CPR Variables

Independent chest compression (CC) variables were total number of chest compressions in two minutes (TCC), mean depth (D), and mean rate (R).

The following are variables in percentage: PCD, the percentage of CC that achieved 50–60 mm goal; PFR, the percentage of CC with full chest recoil; PCR, the percentage of CC delivered at the recommended rate (100–120 per minute). All these percentages were recorded.

Mouth-to-mouth ventilations variables were total number of ventilations (V) during 2 min, estimated total volume of air inflow in ml (TVI) in that time, mean tidal volume in ml per insufflation (MTV), and the percentage of effective airflow ventilation (EV) in which air inflow was enough to cause the chest to rise visibly (from 100 ml onwards).

### 2.4. QCPR Variables

The quality variables were expressed as a percentage of the interventions performed within the standard goal proposed by the 2015 ERC Guidelines. Quality CC (QCC) were those performed with correct hand position, depth between 50 and 60 mm, full chest recoil, and rate between 100 and 120 per minute. A QCC score was calculated using the formula [(PCD + PFR + PCR)/3]. Ventilation quality (QV) was assumed when air inflow was between 500 and 600 ml per mouth-to-mouth insufflation.

Global quality CPR (QCPR) was calculated using the formula [(QCC + QV)/2] [12].

Effective cardiopulmonary resuscitation (ECPR) is the measurement of all quality parameters of resuscitation and ventilation and was calculated using the formula [(EV + QCPR)/2].

The cut-off for quality was established at the arbitrary but generally accepted point of 70% [15].

### 2.5. Rating of Perceived Ability (RPA) and Rating of Perceived Exertion (RPE)

At the end of each test, fishermen were asked to rate their CPR performance through rating of perceived ability (RPA) on a scale of 0–100% and rating of perceived exertion (RPE) during CPR using the modified ten-point Borg scale [16].

### 2.6. Conditions

Data capture was carried out on August 12, 2016, between 16:00 and 20:00 on Rianxo's port (Northwest Spain) beginning at the following GPS location: 42°38′49′′–8°49′30′′. The weather conditions were as follows: air temperature of 27° Celsius, clear sky, wind speed of <1 knot (F0 on Beaufort scale), and calm sea with crests of vitreous appearance and without breaking. APPASPECT GPSSpedd HD, 2.2.3 for IOS (Gujarat, India), registered the movement of the boat and the measure was under 0.5 m in both axes.

### 2.7. Vessels and Sailing

The vessel used was the usual inshore fishing boat in the area, with the following characteristics: arch gross tonnage (GT) of 1.55 T, arch gross register tonnage (GRT) of 2.23 T, total length of 6.7 m, and out-of-board 75-horsepower engine. The boat had enough room on deck for resuscitation of an adult victim.

Sailing was planned and guided by GPS, so the same route during the same time (2 minutes) was guaranteed. Sailing speed was programmed at 10 and 20 sea knots (approximately 20 and 40 km/h) (see Supplementary Video (available here)). One member of the investigation team managed the boat and ensured the same conditions for all subjects.

### 2.8. Statistical Analysis

The sample demographic and anthropometric variables [age, height, weight, and body mass index (BMI)] were expressed by absolute and relative frequencies. The QCPR variables were expressed by central tendency and dispersion measures [mean (standard deviation) (SD)]. The comparison of the dependent variables was performed using ANOVA with Bonferroni and Friedman correction. Statistical differences from parametric variables were determined using Cohen's *d* effect size. Effect sizes with values of 0.2, 0.5, and 0.8 were considered to represent small, medium, and large differences, respectively [17]. From nonparametric variables, the effect size for differences between means was reported using *r* and was interpreted as small when *r* ≥ 0.10, medium when *r* ≥ 0.30, and large when *r* ≥ 0.50 [18]. The process and data analysis were performed using the statistical package SPSS for Windows version 21.0 (SPSS Inc., IBM, USA). A significance level of *p* < 0.05 and a confidence interval of 95% were established.

## 3. Results

Data was available from all participants (20 fishermen); then, 60 CPR 2-minute tests were recorded. Subjects' age was 36 ± 7, weight was 86 kg ± 14, height was 177 cm ± 8, and BMI was 27.3 kg/m^2^ ± 3.

The results of CPR variables are presented in Table 1. When these CPR variables were compared, significant differences were observed for the number of TCC at port versus sailing at 10 knots (158 ± 15 versus 167 ± 18; *p* = 0.01, ES = 1.04) and 20 knots (158 ± 15 versus 171 ± 18; *p* = 0.002, ES = 1.26) (Table 1)

R was quite similar in all conditions, ranging between 115 and 119 compressions per minute (*p* = 0.11)

Although D was significantly better at port versus when sailing at 10 and 20 knots (56 ± 5 versus 60 ± 6 versus 59 ± 7; *p* < 0.05) in all cases, the 2015 international recommendations were fulfilled in all scenarios

During the 2-minute tests, fishermen delivered an average of 10 ventilations, insufflating approximately 5,000 ml of air, with a mean tidal volume ranging from 542 to 601 ml. No significant differences were observed for ventilation variables when scenarios were compared.

**Figure 3:**
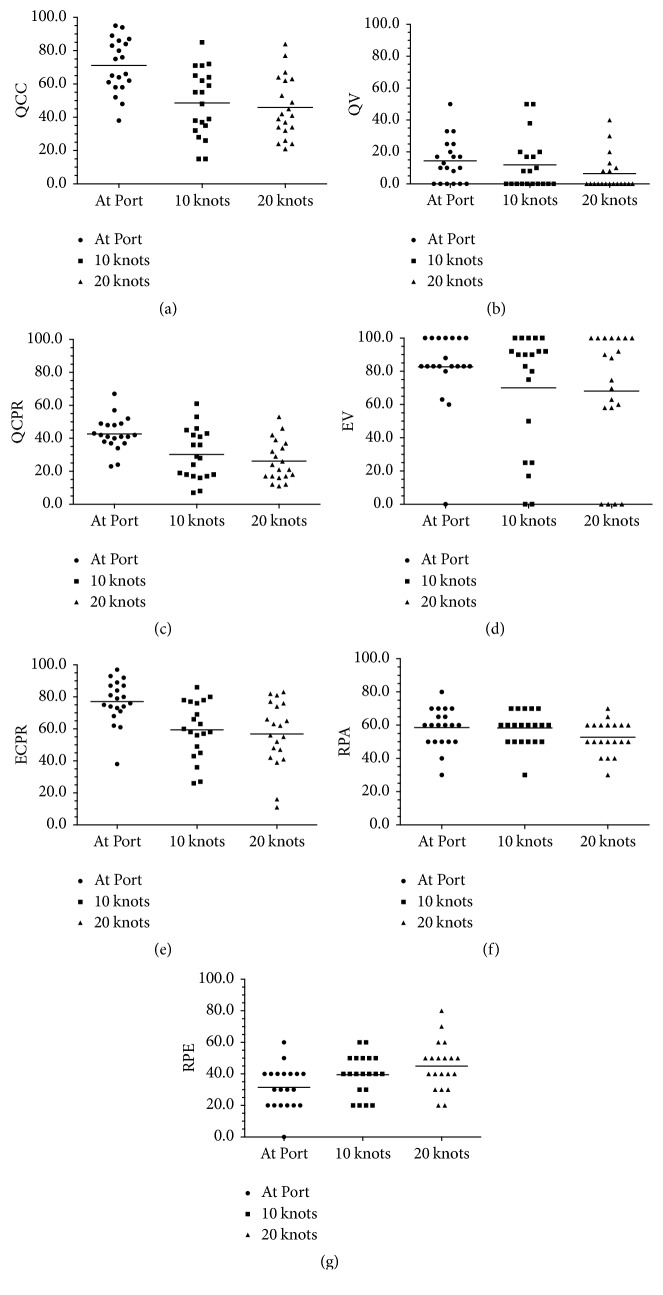
Variables chart.

Data presented in Table 2 and Figure 3 are the values of quality and effective CPR at port and when sailing at 10 and 20 knots. QCC was significantly higher at port (71% ± 16) than while sailing at 10 knots (49% ± 20; *p* = 0.001, ES = 1.42, large) and at 20 knots (46% ± 189; *p* < 0.001, ES = 1.60).

When QCPR was analyzed, higher values were observed at port (43% ± 10) than while sailing at 10 knots (30% ± 15; *p* = 0.01, ES = 1.05) or at 20 knots (26% ± 12; *p* = 0.001, ES = 1.45). 70% of the fishermen exceeded the value of 70%, which has been established as quality criteria [12]. The quality of the ventilation (QV) within the standard goal was very low, obtaining the best value at port (14% ± 14) and the worst value when sailing at 20 knots (6% ± 11), but there were no significant differences in QV at any comparison (*p* > 0.05).

EV was slightly higher at port (83% ± 23) than while sailing at 10 knots (70% ± 36) or at 20 knots (68% ± 38), but not statistically significant (*p* > 0.05).

However, ECPR was significantly higher at port (77% ± 14) than when sailing at 10 knots (59% ± 18; *p* = 0.01, ES = 1.46) or at 20 knots (57% ± 21; *p* = 0.01, ES = 1.44). No significant differences were observed during navigation (at 10 knots: 59% ± 18, at 20 knots: 57% ± 21; *p* = 1.00, ES = 0.35).

Fishermen found it more difficult to perform CPR at 20 knots (53% ± 10 versus 59% ± 12; *p* = 0.02, ES = 0.50) than at port. For the rest of the comparisons, no significant differences were observed (RPA at port: 59% ± 12, RPA when sailing at 20 knots 53% ± 10; *p* = 0.02, ES = 0.50). However, when compared to the baseline, the perceived effort increased with sailing speed (RPE at port: 3 ± 1, RPE when sailing at 10 knots: 4 ± 1; *p* = 0.01, ES = 0.57; RPE when sailing at 20 knots: 5 ± 2; *p* < 0.001, ES = 0.72; RPE when sailing at 10 knots: 4 ± 1; RPE when sailing at 20 knots: 5 ± 2; *p* = 0.01, ES = 0.55).

## 4. Discussion

Our study, which evaluated CPR quality by fishermen at work on a small fishing boat, showed that CPR is feasible in such boats while navigating to port, but QCPR decreased as the navigation speed increased.

Groups identified as being at a high risk of experiencing medical emergencies or injuries as fishermen should be targeted in training and prevention initiatives [3, 19]. There is evidence that training lay people improves their willingness to undertake CPR in a real situation [20].

The fishermen, after a brief review session, were more likely to perform QCPR at port than at 10 and 20 knots, respectively. Most of the fishermen exceeded a value of 70% in QCC; these values of QCC at port were similar to those made by other healthcare and nonhealthcare groups (lifeguards [21, 22], police [23], lay rescuers [24], teachers [25], and healthcare providers [26, 27]). However, when CPR was performed while navigating, QCPR decreased as we observed in the literature [7–9].

TCC was observed to increase while navigating. D and R increased but with a minor effect. The same was observed in a similar study [6] and in studies evaluating fatigue [21, 22].

The results observed while navigating are interesting; TCC and D increased, respectively, with higher speed; this could be due to a worse control of strength and proprioception when performing CPR in an unstable environment. Previous studies evaluating CC quality in different simulated transports observed that instability was a determining factor [9, 28, 29]. Sailing even in calm waters produces instability to the rescuers, a fact that contributes to the decrease of CC quality. A previous study that evaluated continuous CC quality while sailing with tailwind or headwind obtained similar results [9].

Fighting cerebral hypoxia is essential for drowning victims [30]. Fishermen were able to provide a correct tidal volume, obtaining similar values to lifeguards ventilating mouth to mouth [31].

Borg's scale is often used in clinical papers to estimate the magnitude of the perceived effort during out-of-hospital CPR maneuvers [6, 32–34]. Fatigue is a limitation to CPR ability; therefore, the RPE can offer relevant information to support which physical conditions the rescuer must have or how long they will be able to maintain a good technique. In our study, fishermen reported a harder effort feeling as speed increased. Similar rating was found in studies where professional lifeguards performed CPR while sailing at 10 knots [6] or during simulated rescue helicopter flights [29].

Self-assessment is a mechanism that helps improve practical learning patterns and is a way of changing self-behaviour [34]. Currently, there are not many studies on self-assessment of CPR skills, but there is a coincidence in overestimation of abilities [34, 35] as well as in overestimation of CPR success in aquatic environments [36]. Curiously, in our research, we observed the opposite: fishermen underestimated their ability to perform CPR, especially at port where they had greater proprioceptive control.

Maritime rescues are a common and a worldwide situation. It is known that fishing is one of the most dangerous occupations and prompt and effective actions by trained rescuers make the difference between life and death. These findings are important in supporting the notion that fishermen must be trained regularly and showed that they must start early CPR while navigating at higher speed better than slowly to reach as soon as possible the nearest land in an emergency situation at aquatic environment.

## 5. Limitations

Some limitations that must be considered to correctly read our results include the weather and sea conditions and the emotional and lifestyle factor; the use of a manikin in a simulated situation of emergency may cause bias.

It is important to consider the sequence of testing which may influence the results because of fatigue (negative) or learning (positive). By performing the navigation tests with short pauses, fisherman can learn and become better although the improvement would be minimal as they do not have visual feedback and would become fatigued.

## 6. Conclusions

CPR trained and refreshed fishermen are able to perform good quality CPR (chest compressions and mouth-to-mouth ventilations) on manikins both on land and while sailing at relatively low (10 knots) and high (20 knots) navigating speeds.

Our results support the notion that, in the case of drowning or cardiac arrest on a small fishing boat, in calm sea conditions, fishermen should immediately start basic CPR and maintain CPR while navigating at a relatively high speed (around 20 knots) to the nearest land, where CPR would be continued with advanced procedures by expert personnel.

## Figures and Tables

**Figure 2:**
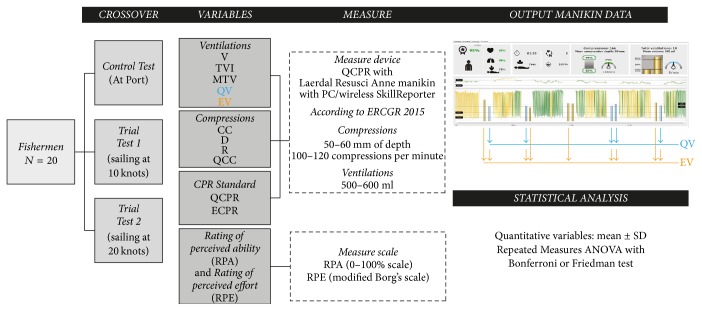
Flowchart design.

**Table 1:** CPR variables (compressions and ventilations).

	*At port *		*At 10 knots *		*At 20 knots *		*At port versus 10 knots*			*At port versus 20 knots*			*10 knots versus 20 knots*		
	Mean (DE)	95% IC	Mean (DE)	95% IC	Mean (DE)	95% IC	*p* value	ES	Rating	*p* value	ES	Rating	*p* value	ES	Rating
*Compressions*															
TCC^*∗*^	**158** (15)	151–165	**167** (18)	159–176	**171** (18)	163–180	0.01	1.04	Large	0.002	1.26	Large	0.09	0.74	Medium
D^*∗∗*^	**56** (5)	54–58	**60** (6)	57–63	**59** (7)	56–62	<0.001	0.69	Large	0.003	0.65	Large	0.06	0.30	Medium
R^*∗∗*^	**115** (16)	108–123	**119** (11)	114–124	**115** (27)	102–128	0.11	0.44	Medium	0.11	0.40	Medium	0.11	0.26	Small
*Ventilations*															
V^*∗*^	**11** (2)	10-11	**10** (2)	10-11	**10** (1)	10-11	1.00	0.14	Small	0.56	0.43	Small	1.00	0.26	Small
TVI^*∗∗*^	**4939** (1927)	4038–5841	**5088** (3211)	3585–6591	**5233** (4066)	3330–7136	0.66	0.06	Trivial	0.49	0.08	Trivial	0.64	0.04	Trivial
MTV^*∗∗*^	**542** (81)	457–627	**601** (267)	476–726	**571** (405)	381–760	0.25	0.22	Small	0.82	0.15	Small	0.64	0.10	Small

^*∗*^Bonferroni test. ^*∗∗*^Friedman test. TCC: total number of chest compressions (during two minutes). D: depth of chest compression (mm). R: chest compressions rate (per minute). V: ventilations (during two minutes). TVI: total volume inflated (ml during two minutes). MTV: mean tidal volume (ml per ventilation).

**Table 2:** Quality and effective CPR. Perceived exertion and quality variables.

	*At port*		*At 10 knots*		*At 20 knots*		*At port versus 10 knots*			*At port versus 20 knots*			*10 knots versus 20 knots*		
	Mean (DE)	95% IC	Mean (DE)	95% IC	Mean (DE)	95% IC	*p* value	ES	Rating	*p* value	ES	Rating	*p* value	ES	Rating
*Quality maneuvers*															
QCC^*∗*^	**71**(16)	63–79	**49** (20)	39–58	**46** (18)	37–54	0.001	1.42	Large	<0.001	1.60	Large	0.47	0.46	Small
QV^*∗∗*^	**14** (14)	8–21	**12** (17)	4–20	**6** (11)	1–12	0.59	0.15	Small	0.13	0.47	Medium	0.17	0.33	Medium
QCPR^*∗*^	**43** (10)	38–47	**30** (15)	23–37	**26** (12)	20–32	0.01	1.05	Large	0.001	1.45	Large	0.27	0.56	Medium
*Effective maneuvers*															
EV^*∗∗*^	**83** (23)	72–94	**70** (36)	53–87	**68** (38)	50–85	0.47	0.23	Small	0.07	0.43	Medium	0.59	0.22	Small
ECPR^*∗*^	**77** (14)	71–83	**59** (18)	51–67	**57** (21)	47–66	0.01	1.46	Large	0.01	1.44	Large	1.00	0.35	Small
*Perceived variables*															
RPA^*∗∗*^	**59** (12)	53–64	**58** (10)	54–63	**53** (10)	48–57	1.00	0.10	Small	0.02	0.50	Large	0.06	0.51	Large
RPE^*∗∗*^	**3** (1)	3-4	**4** (1)	3–5	**5** (2)	4-5	0.01	0.57	Large	<0.001	0.72	Large	0.01	0.55	Large

^*∗*^Bonferroni test. ^*∗∗*^Friedman test. QCC: chest compression quality (percentage of TCC with 50 and 60 mm of depth, with full chest recovery, keeping a rate between 100 and 120 compressions per minute). QV: quality of ventilations in percentage with a tidal volume between 500 and 600 ml. QCPR: quality of CPR, calculated with the formula [(QCC + QV)/2]. EV: effective ventilation with visible air intake (from 100 ml onwards) in percentage. ECPR: effectiveness of CRP in percentage, calculated with the formula [(QCC + EV)/2]. RPA: rating of perceived ability (in percentage). RPE: rating of perceived exertion (modified 10-point Borg's scale).

## Supplementary Materials

Supplementary Materials A video of how the research was done is included in this manuscript as a supplementary file.
